# Recent Patents of Interest to Dentists

**Published:** 1907-04

**Authors:** 


### PATENTS.

### RECENT PATENTS OF INTEREST TO DENTISTS.

841946. Tooth-brush, Fred Downing, Decatur, Illinois.
841962. Dental flask-press, James F. Hardy, New York, N. Y.
842112. Dental plugger, Safford G. Perry, NewYork, N. Y.
842357. Filling teeth, Hamilton F. Strong, Cleveland. Ohio.
843273. Dental bur and excavator, Willy Homann, Dusseldorf, Germany.

844079. Dental matrix, Ellsworth Armstrong, Collingdale, Pa.
844181. Dental floss holder, Charles M. Overbaugh, Clarion, Iowa.

Copies of above patents may be obtained for fifteen cents each by addressing John A. Saul, Solicitor of Patents, Fendall Building, Washington, D. C.

